# A novel magnetic resonance imaging index to evaluate ovarian reserve in endometrioma patients: the ovary to endometrioma volume index

**DOI:** 10.3389/fmed.2026.1760689

**Published:** 2026-04-14

**Authors:** Mesut Önal, Adem Kocaman, Yunus Katırcı, İlkay Çamlıdağ, Hüseyin Ekici, Abdülkadir Bakay

**Affiliations:** 1 Department of Obstetrics and Gynecology, Faculty of Medicine, Ondokuz Mayis University, Samsun, Türkiye; 2 Department of Histology and Embryology, Faculty of Medicine, Ondokuz Mayis University, Samsun, Türkiye; 3 Department of Radiology, Faculty of Medicine, Ondokuz Mayis University, Samsun, Türkiye

**Keywords:** AMH4, endometrioma, magnetic resonance imaging, ovarian reserve, volumetric index

## Abstract

**Background:**

Ovarian endometrioma is a common form of endometriosis associated with chronic pelvic pain, infertility, and diminished ovarian reserve. Although anti-Müllerian hormone (AMH) is widely used to evaluate ovarian reserve, it does not reflect structural ovarian changes. To introduce and assess the Ovary to Endometrioma Volume Index (OEVI), calculated from magnetic resonance imaging (MRI), as noninvasive marker of ovarian reserve in women with endometrioma.

**Methods:**

Fifty women of reproductive age with ovarian endometrioma were included. Patients with menopause, primary ovarian failure, or prior endometrioma surgery were excluded. Serum AMH, follicle stimulating hormone (FSH), luteinizing hormone (LH), and cancer antigen 125 (Ca-125) were measured. Ovarian and endometrioma volumes were quantified on pelvic MRI, and OEVI was calculated as total ovarian volume divided by total endometrioma volume. Spearman correlation and multivariable regression analyses were performed.

**Results:**

OEVI showed a positive correlation with AMH (*ρ* = 0.332) and negative correlations with FSH (ρ = −0.306) and LH (ρ = −0.422). In exploratory multivariable analysis, AMH showed a positive association with OEVI (*β* = 0.337, *p* = 0.030), whereas Ca-125 (*β* = −0.018, *p* = 0.022) and LH (*β* = −0.141, *p* = 0.035) demonstrated inverse associations. The regression model explained 42% of OEVI variability. The regression model explained 42% of OEVI variability.

**Conclusion:**

OEVI demonstrates significant associations with established hormonal markers and may represent a promising structural adjunct in ovarian reserve assessment. Its association with AMH and inverse relationship with gonadotropins suggest that OEVI reflects both structural and functional ovarian capacity and may support individualized clinical management and fertility counseling.

## 1 Introduction

Endometriosis is a chronic gynecological disorder characterized by the ectopic presence of endometrial glands and stroma, affecting approximately 6–10% of women of reproductive age and contributing to pelvic pain, infertility, and a significant decline in quality of life (1–3). Among its subtypes, ovarian endometrioma represents a distinct clinicopathological entity with an estimated prevalence of 17–44% in women diagnosed with endometriosis (4–6). The pathogenesis of ovarian endometrioma is multifactorial and incompletely understood, encompassing retrograde menstruation, oxidative stress, aberrant iron metabolism, and inflammatory activation within the ovarian microenvironment (7–10). Iron overload secondary to cyclical hemorrhage in endometriotic cysts promotes lipid peroxidation and cellular injury, which results in cortical fibrosis, follicular loss, and diminished oocyte quality (7, 11, 12). These mechanisms contribute to a measurable reduction in ovarian reserve, as reflected by decreased antral follicle count (AFC) and serum anti-Müllerian hormone (AMH) levels in affected women compared with healthy counterparts (13–15). Moreover, even before surgical excision, endometrioma itself exerts mechanical and biochemical effects on the ovarian cortex, accelerating the age-related decline in follicular reserve and compromising assisted reproductive technology (ART) outcomes (13, 14, 16–18).

Biochemical parameters such as AMH are widely recognized as the most sensitive hormonal marker for evaluating follicular capacity, which provides cycle-independent and reproducible results (19–21). However, AMH alone does not reflect the morphologic alterations of the ovarian tissue caused by endometriotic infiltration or compressive effects, which are critical for predicting the reproductive potential (9, 15, 22). Imaging modalities, including ultrasonography and magnetic resonance imaging (MRI), have therefore gained importance in the morphometric evaluation of endometrioma. Volumetric indices such as the ultrasonography-based Affected Ovary Relative Volume (AORV) proposed by Cosma et al. have shown significant correlations with AMH and AFC in unilateral endometrioma (23). More recently, MRI-based techniques have expanded from structural to functional applications, with Zhang et al. using R2 relaxometry to quantify iron deposition, which provided a non-invasive insight into oxidative stress-related tissue injury (24).

While serum anti-Müllerian hormone (AMH) levels are widely used as a biochemical marker, they do not reflect structural ovarian changes. Thus, this study aims to introduce and evaluate a novel magnetic resonance imaging (MRI)-based volumetric parameter -the Ovary-to-Endometrioma Volume Index (OEVI)- as a potential non-invasive imaging biomarker for assessing ovarian reserve. The development of such an MRI-derived index may enhance the precision of ovarian reserve assessment and assist in individualized management of endometrioma patients.

## 2 Methods

This study was conducted in the Obstetrics and Gynecology Department of Ondokuz Mayıs University Faculty of Medicine, Türkiye. The related ethical approval was obtained from the institutional review board (Approval No: OMU-2024/114). Patients admitted to the department with a complaint of pelvic pain or infertility and diagnosed with ovarian endometrioma were included in the study. Patients with menopause, primary ovarian failure, or previous endometrioma surgery were excluded. Following recruitment, all the patients underwent biochemical analyses, including Ca-125, follicle-stimulating hormone (FSH), luteinizing hormone (LH), and AMH, and pelvic magnetic resonance imaging (MRI) assessment for volumetric measurements of the ovaries and endometrioma lesions. Patients with previous ovarian surgery for endometrioma, primary ovarian insufficiency, or menopausal status were excluded to minimize confounding effects on ovarian reserve assessment. Both unilateral and bilateral endometriomas were included, and volumetric calculations were performed using the sum of right and left ovarian and endometrioma volumes.

### 2.1 AMH assay procedure

The AMH test is performed with the Roche Hitachi cobas® 6,000/cobas e601 device using the electrochemiluminescence immunoassay (ECLIA) method as previously described (20). The test process is carried out on the basis of the sandwich principle, based on the streptavidin-biotin- technology. Antigen–antibody complexes were detected by the electrochemiluminescence method within 18 min of analysis time. On the other hand, AMH was detected in the range of 0.01 to 23 ng/mL using 50 μL of the serum sample.

### 2.2 MRI assessments

All the pelvic MRI examinations were performed using a 3 T magnet (Philips, Ingenia, Netherlands). Patients were scanned in the supine position, and the following protocol was used for all the patients. Axial, coronal, and sagittal T2-weighted images without fat saturation, axial T1-weighted images without fat saturation, and axial dynamic contrast-enhanced T1-weighted images with fat saturation with IV Gadolinium were obtained. All the MR images were transferred to an Osirix imaging software for radiological evaluation. Volumetric analyses of the ovaries and the endometriomas were performed by a board-certified radiologist with 8 years of experience in abdominal radiology blinded to the study and clinical information of the patients. Axial T2 weighted images without fat saturation were used for analysis. In these images, the borders of normal-appearing ovaries were manually traced on each slice, they were visualized on both sides, and surface areas were measured in mm^2^. Afterward, the edges of each ovarian lesion suggestive of endometrioma based on MRI findings were manually traced in the same fashion, and the surface areas were measured. To calculate the volumes of the ovaries and the endometriomas in mm^3^, the surface areas were summed and multiplied by the slice thickness based on the Cavalieri principle. Volumetric measurements were performed using a standardized manual segmentation protocol based on the Cavalieri principle. All measurements were conducted by a board-certified radiologist blinded to clinical and hormonal data to reduce measurement bias.

### 2.3 Volumetric calculations

Using the volumetric measurements obtained from MRI assessments, a novel index was calculated. Namely, the ovary to endometrioma volume index (OEVI) was calculated as follows:


$$OEVI=\frac{Total\;ovary\;volume}{Total\;endometrioma\;volume}\;$$

The total ovary and endometrioma volumes were calculated by summing the relevant volumes on the left and right sides:


$$Total\;ovary\;volume=Ovary\;volume_{Right}+Ovary\;volume_{Left}$$

$$\begin{array}{c} Total\;endometrioma\;volume=Endometrioma\;volume_{Right}\;\\ +Endometrioma\;volume_{Left} \end{array}$$

In unilateral cases, total endometrioma volume corresponded to the affected ovary, whereas contralateral endometrioma volume was considered zero.

### 2.4 Statistical methods

**Figure 1:**
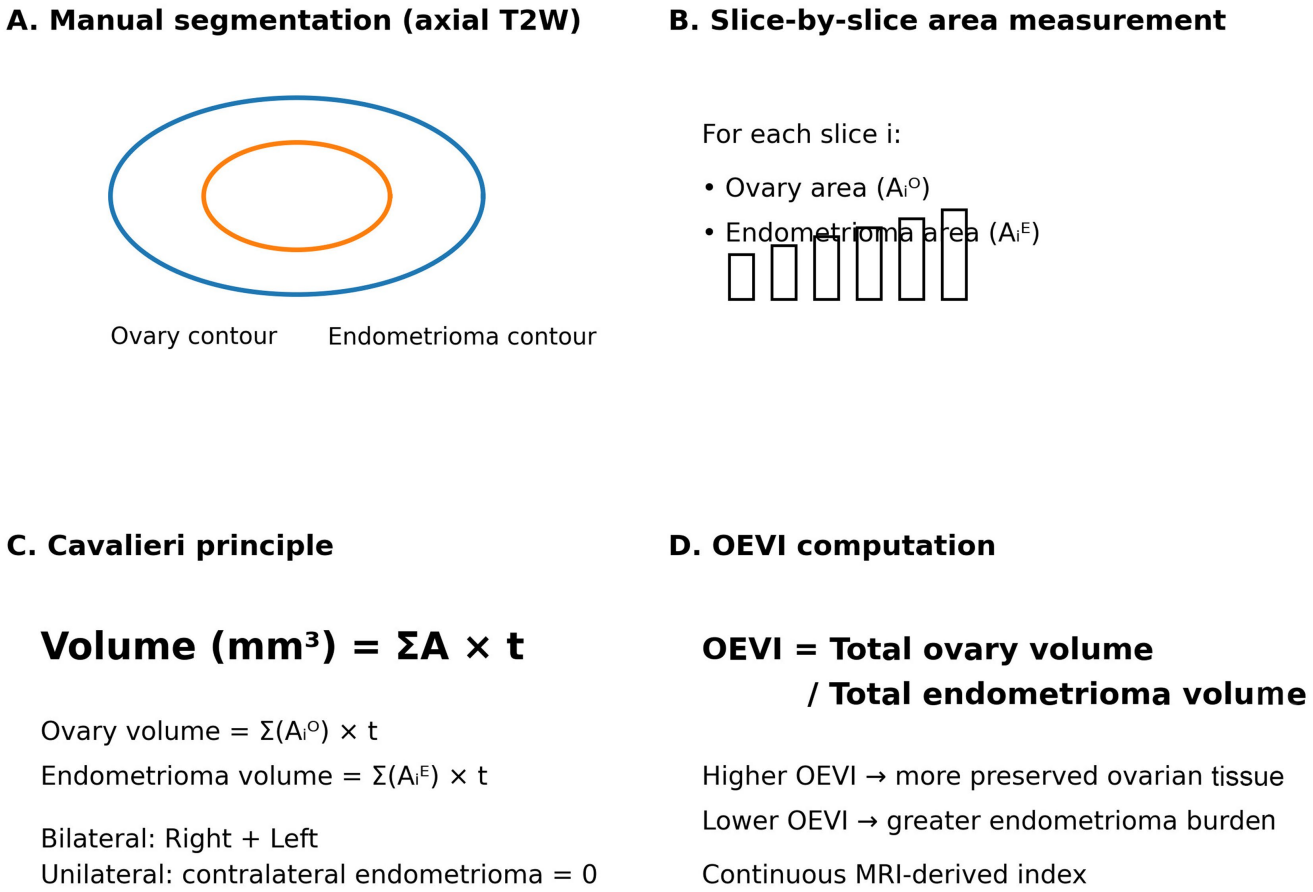
Flowchart.

**Figure 2:**
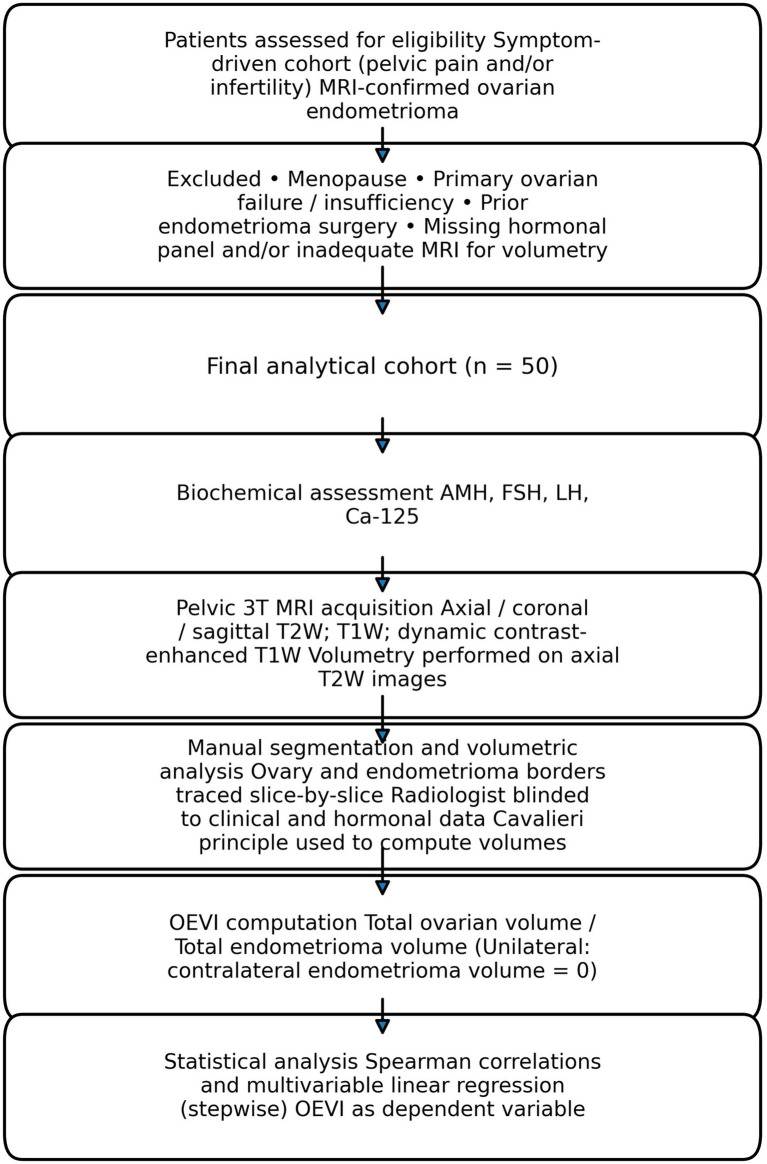
Schematic illustration of OEVI calculation from pelvic MRI volumetry. **(A)** Manual segmentation of ovarian and endometrioma contours on axial T2-weighted MRI images. **(B)** Slice-by-slice area measurement of ovarian (A_i_O) and endometrioma (A_i_E) regions. **(C)** Volume calculation using the Cavalieri principle, with summation of slice areas multiplied by slice thickness. **(D)** Computation of the Ovary-to-Endometrioma Volume Index (OEVI) as the ratio of total ovarian volume to total endometrioma volume.

Descriptive statistics were presented using median and interquartile range (IQR: 25th-75th percentile) for continuous variables. The correlation between biochemical parameters and volumetric measurements was analyzed using Spearman’s nonparametric correlation analysis. Variables with clinical relevance (AMH, Ca-125, FSH, LH) were entered simultaneously into a multivariable linear regression model. Due to missing data, regression analysis was performed using complete-case analysis. A *p*-value<0.05 was considered statistically significant. All the analyses were performed in SPSS 28 (IBM Inc., Armonk, NY, USA) (Figures 1, 2).

## 3 Results

A total of 50 patients were included in the study. The median age was 33 years [30–37], and the median body mass index (BMI) was 21.7 kg/m^2^ [20.3–22.7]. The median serum AMH level was 0.80 ng/mL [0.57–1.70], while median Ca-125 concentration was 40.86 U/mL [23.5–57.2]. Median FSH and LH level were 12.00 mIU/mL [6.4–27.5] and 9.30 IU/L [6.3–14.0], respectively. Regarding volumetric measurements, the median total ovarian volume was 18.37 mm^3^ [13.42–26.55], and the median total endometrioma volume was 33.00 mm^3^ [13.40–79.71]. The Ovary-to-Endometrioma Volume Index (OEVI) had a median value of 0.45 [0.25–0.94], indicating substantial interindividual variability in the structural balance between ovarian tissue and endometrioma burden. Endometriomas involved one or both ovaries across the study population. OEVI values demonstrated a wide distribution, reflecting heterogeneous structural involvement of ovarian tissue among patients (Tables 1–3).

**Table 1:** Demographic, biochemical and volumetric measurements.

Variables	**Median [IQR]**
Age (years)	33 [30–37]
BMI (kg/m^2^)	21.7 [20.3–22.7]
Ca-125 (U/ml)	47.43 [12.6–122.6]
FSH (mlU/ml)	16.42 [3.1–42]
LH (IU/L)	9.84 [2.8–20]
AMH (ng/ml)	1.32 [0.1–6.2]
Volumetric measurements	
Ovary volume (mm^3^)	5.31 [0–14.08]
Endometrioma volume (mm^3^)	4.93 [0–68.9]
Ovary/Endometrioma index	1.70 [0.03–2.91]

**Table 2:** Correlations between biochemical and volumetric measurements.

Predictor	**OEVI**	**AMH (ng/ml)**	**Ca-125 (U/ml)**	**FSH (mIU/ml)**
AMH (ng/ml)	0.332 (0.105)			
Ca-125 (U/ml)	0.163 (0.518)	0.237 (0.344)		
FSH (mIU/ml)	−0.306 (0.136)	0.057 (0.786)	−0.031 (0.903)	
LH (IU/L)	−0.422 (0.036)	0.202 (0.332)	−0.309 (0.212)	0.732 (<0.001)

**Table 3:** Independent determinants of the OEVI in a multivariable regression model.

Predictor	B	SE	95% CI for B	p
Intercept	1.639	0.536	0.481–2.797	0.009
AMH (ng/ml)	0.337	0.138	0.039–0.635	0.030*
Ca-125 (U/ml)	−0.018	0.007	−0.033 – −0.003	0.022*
FSH (mIU/ml)	0.045	0.022	−0.002 – 0.093	0.060
LH (IU/L)	−0.141	0.060	−0.270 – −0.012	0.035*

Model Summary: *R*^2^ = 0.419, Adjusted *R*^2^ = 0.240, *F*(4, 13) = 2.345, *p* = 0.109. β = standardized coefficient. * Indicates statistical significance (*p* < 0.05).

Normality tests indicated that OEVI, AMH, Ca-125, and FSH were not normally distributed (Shapiro–Wilk *p* < 0.005 for OEVI, AMH, FSH; *p* = 0.024 for Ca-125), whereas LH showed a normal distribution (*p* = 0.3436). Spearman correlation analysis revealed a positive correlation between OEVI and AMH (*ρ* = 0.332), while OEVI correlated negatively with LH (ρ = −0.422) and FSH (ρ = −0.306). No significant correlation was observed between OEVI and Ca-125 (ρ = 0.163).

Multiple linear regression analysis was performed with OEVI as the dependent variable and AMH, Ca-125, FSH as well as LH as independent variables. The overall model explained 42% of the variance in OEVI (R^2^ = 0.419). AMH was identified as a significant independent predictor” is replaced with “Within the multivariable model, AMH showed a significant positive association with OEVI (*β* = 0.337, 95% CI 0.039 0.635, *p* = 0.030). Conversely, Ca-125 (*β* = −0.018, 95% CI –0.033 to –0.003, *p* = 0.022) and LH (*β* = −0.141, 95% CI –0.270 to −0.012, *p* = 0.035) were significant negative predictors. FSH demonstrated a borderline positive effect (*β* = 0.045, *p* = 0.060). Residuals from the regression model were normally distributed (Shapiro–Wilk *W* = 0.939, *p* = 0.277), and multicollinearity diagnostics indicated acceptable variance inflation factors (VIFs < 3.8 for all predictors). These findings suggest that OEVI reflects not only hormonal ovarian reserve but also the relative structural burden of endometrioma on ovarian tissue.

These findings suggest that AMH is independently associated with OEVI, while Ca-125 and LH are inversely related, highlighting their potential relevance in ovarian-endometrial volumetric assessment.

## 4 Discussion

This study has evaluated whether a volumetric index calculated using total ovarian volume and endometrioma volume can be used to measure ovarian reserve in patients with endometrioma. The findings were compared with anti-Müllerian hormone (AMH) levels, which represent the most accepted biochemical parameter for ovarian reserve assessment. In addition, the correlation with cancer antigen-125 (Ca-125) levels was analyzed. The results demonstrated a significant positive correlation between the Ovary-to-Endometrioma Volume Index (OEVI) and AMH, whereas OEVI showed negative correlations with follicle-stimulating hormone (FSH) and luteinizing hormone (LH). These findings indicate that the volumetric relationship between the endometrioma and ovarian tissue reflects functional ovarian capacity. Rather than serving as a dichotomous diagnostic marker, OEVI should be interpreted as a continuous MRI-derived index reflecting the balance between preserved ovarian tissue and endometrioma burden. The observed associations with AMH and gonadotropins support its role in individualized risk stratification rather than threshold-based classification.

Ovarian endometrioma is a frequent manifestation of endometriosis, contributing to chronic pelvic pain, dysmenorrhea, and infertility (1, 2, 4). Previous studies have established that the presence of endometrioma adversely affects ovarian reserve due to follicular damage, cortical fibrosis, and inflammatory mechanisms (7, 9, 13, 14). In the present study, the OEVI was found to be positively associated with AMH and inversely associated with gonadotropins, suggesting that reduced ovarian parenchymal volume relative to endometrioma mass is accompanied by hormonal evidence of follicular depletion.

Our findings are consistent with those of Uncu et al., who reported significantly decreased AMH and antral follicle counts (AFC) in women with endometrioma compared with healthy controls and demonstrated a further postoperative decline following cystectomy (13). Similarly, Kasapoğlu et al. introduced the concept of “endometrioma-related reduction in ovarian reserve (ERROR)” and confirmed a longitudinal decrease in AMH after endometrioma excision (14). In agreement with these results, our study shows that even before surgical intervention, an increased endometrioma burden (lower OEVI) is associated with lower AMH levels, indicating a preoperative functional decline in ovarian reserve.

The pathophysiological mechanisms underlying this reduction have been attributed to oxidative stress and iron overload. Kitajima et al. demonstrated that chronic inflammation, iron accumulation, and stromal fibrosis cause follicular loss and impaired oocyte maturation in endometriotic ovaries (9). Likewise, Liu et al. emphasized that iron metabolism abnormalities in endometriotic lesions contribute to cytotoxic oxidative injury and tissue degeneration (7). Yoshimoto et al. further showed that cyst fluid iron-related compounds may even predict malignant transformation (11), while Woo et al. revealed that ferritin upregulation in endometriotic tissue enhances the migratory phenotype of endometrial cells (8). These biochemical findings conceptually support the volumetric changes captured by OEVI in our study, since a larger endometrioma-to ovary ratio likely corresponds to greater oxidative and fibrotic injury. Our findings indicate that volumetric encroachment of endometrioma within the ovary may contribute to functional decline even before surgical intervention.

The association between lesion volume and diminished reserve is well-documented. Jiang and Nie and Yılmaz Hanege et al. both noted that endometrioma size and duration are inversely proportional to ovarian reserve (4, 15). Similarly, Karadağ et al. demonstrated that larger and bilateral endometriomas significantly reduce AMH and AFC compared to unilateral or smaller lesions (22). Our results are parallel with these observations, confirming that volumetric encroachment within the ovary -quantified as a decreased OEVI correlates with hormonal markers of reduced follicular capacity.

From a functional perspective, the positive correlation between OEVI and AMH aligns with prior evidence that AMH is the most stable and cycle-independent biomarker of ovarian reserve (19–21) Anderson et al. and the ASRM Practice Committee emphasized that AMH measurement provides high clinical reliability, while La Marca et al. and Seifer et al. demonstrated age-related decline patterns (18–21). In this context, our regression analysis revealed that OEVI showed a significant association with AMH within the multivariable model, suggesting that OEVI could complement hormonal evaluation by incorporating structural information on ovarian integrity.

Previous imaging-based studies provide additional support for our approach. Cosma et al. proposed the “Affected Ovary Relative Volume” (AORV) ratio as an ultrasonographic predictor of ovarian reserve in unilateral endometrioma, reporting that smaller AORV values correlate with lower AMH (23). Our MRI based OEVI reflects a conceptually similar relationship, yet it offers greater accuracy in delineating cyst margins and ovarian parenchyma. MRI’s superior soft-tissue contrast enhances the precision of volumetric calculations, as also shown by Noventa et al., who reported MRI’s superiority over transvaginal ultrasonography in detecting endometriotic lesions (25). Therefore, OEVI may represent an evolution of the AORV concept with improved anatomical resolution.

The influence of inflammation and oxidative stress was also established by Sánchez et al. and Di Emidio et al., who demonstrated increased markers of oxidative and carbonyl stress in ovarian cortex adjacent to endometriomas, leading to granulosa cell apoptosis and oocyte dysfunction (10, 12). The volumetric reduction observed in our study correspondingly reflects this same destructive microenvironment, linking structural deformation with cellular oxidative injury.

In parallel, iron-related imaging biomarkers have recently gained attention. Zhang et al. used magnetic resonance imaging (MRI) R2 mapping to quantify iron deposition in ovarian endometriomas non-invasively, proposing it as a surrogate of oxidative stress and tissue damage (24). Integrating such advanced MRI metrics with volumetric indices like OEVI could provide a comprehensive morphofunctional assessment of ovarian reserve, combining structural and biochemical insights.

Dyndar et al. recently showed that women with endometrioma exhibit markedly reduced reproductive potential, characterized by lower AMH and higher gonadotropin levels, which is consistent with our findings (26). Yin et al. (2024) further demonstrated, using three-dimensional transvaginal ultrasound (3D-TVUS), that ovarian volume and AMH are directly correlated in both adenomyosis and endometriosis, supporting the volumetric-functional association confirmed by our MRI-based results (27). Collectively, these findings substantiate that quantitative volumetric indices such as OEVI, being biologically meaningful and clinically relevant in assessing ovarian reserve.

Although our findings are robust, several limitations should be considered. The first aspect is related to the cross-sectional design precludes longitudinal evaluation of temporal changes in OEVI and AMH during disease progression or after treatment. Second, the absence of a healthy control group limits our ability to define a cut-off value for impaired ovarian reserve. Third, potential confounding factors such as age, disease bilaterality, and coexisting adenomyosis were not separately analyzed. Consequently, future studies should incorporate larger cohorts, age stratification, and combined imaging biomarkers such as MRI R2 mapping t refine diagnostic precision and establish prognostic thresholds for OEVI.

In conclusion, the OEVI represents a promising, non-invasive MRI-based biomarker for evaluating ovarian reserve in women with endometrioma. Its positive association with AMH and negative correlation with gonadotropins align with established endocrine and morphologic evidence of diminished ovarian function. OEVI may thus serve as a valuable adjunct in clinical decision-making, which can facilitate individualized management strategies, fertility counseling as well as the monitoring of endometrioma-related ovaria damage.

### 4.1 Limitations

This study has several limitations. First of all, the cross-sectional design precludes assessment of longitudinal changes in OEVI and AMH following disease progression or treatment. Moreover, the absence of a healthy control group limits comparative interpretation and the establishment of diagnostic thresholds. Additionally, potential confounders such as age, bilaterality, and coexisting adenomyosis were not separately analyzed. Additionally, multivariable regression analysis was conducted in a reduced complete-case subset (*n* = 18), which may have limited statistical power and increased the risk of model overfitting. Therefore, future prospective studies with larger cohorts and multimodal imaging approaches are warranted to validate these findings.

## 5 Conclusion

The Ovary-to-Endometrioma Volume Index (OEVI) is a novel, MRI-based, non-invasive biomarker that reflects ovarian reserve in women with endometrioma. Its positive correlation with AMH and negative associations with gonadotropins indicate that it effectively represents the functional capacity of the ovary. By providing an anatomical complement to biochemical markers, OEVI may assist in preoperative evaluation, fertility counseling, and individualized management. Further prospective studies integrating OEVI with advanced MRI techniques are required to validate its clinical and prognostic utility.

## Data Availability

The raw data supporting the conclusions of this article will be made available by the authors, without undue reservation.
